# Herbarium of the University of Malaga (Spain): Vascular Plants Collection

**DOI:** 10.3897/phytokeys.26.5396

**Published:** 2013-09-27

**Authors:** José García-Sánchez, Baltasar Cabezudo

**Affiliations:** 1 Herbarium curator.University of Málaga, Servicios Centrales de Apoyo a la Investigación (SCAI). Bulevar Louis Pasteur, 33. Campus de Teatinos, 29071, Málaga, Spain; 2 Scientific Manager.University of Málaga, Departament of Plant Biology (Botany Area), Faculty of Sciences, University of Málaga. Campus Teatinos s/n., 29071, Málaga, Spain

**Keywords:** MGC, MGC Herbarium, University of Malaga (SCAI), Cormophyta, Spermatophyta, Pteridophyta, Compositae, Leguminosae, Gramineae, Labiatae, Caryophyllaceae, Cruciferae, Scrophulariaceae, Cistaceae, Umbelliferae, Liliaceae, Western Mediterranean Region, Spain, Southern Spain, Southern Iberian Peninsula, Iberian Peninsula, Andalusia (Spain), Malaga province (Spain), Northern Morocco

## Abstract

The herbarium of University of Málaga (MGC Herbarium) is formed by four biological collections. The vascular plants collection (MGC-Cormof) is the main collection of the herbarium. MGC-Cormof dataset aims to digitize and publish data associated with over 76.000 specimens deposited in the collection, of which 97.2% of the specimens are identified at species level. Since 2011, the University of Malaga’s Central Research Service (SCAI) has been responsible for maintaining the herbariums and the dataset. The collection is growing continuously, with an annual intake of about 1.500 specimens. Nearly 96% of the collection is digitized, by Herbar v3.7.1 software (F. Pando et al. 1996–2011), making over 73.000 specimens accessible through the GBIF network (http://data.gbif.org/datasets/resource/8105/). At present, 247 families and 8.110 taxa, distributed in angiosperms (93.97%), ferns and fern allies (4.89%) and gymnosperms (1.14%), constitute the MGC-Cormof collection. The families and genera best represented in the collection are Compositae, Leguminosae, Gramineae, Labiatae, Caryophyllaceae, Teucrium, Silene, Asplenium, Linaria and Quercus. Most of the specimens are from the Western Mediterranean Region, fundamentally Southern Spain (Andalusia: 82% of specimens) and Northern Morocco (2.17%). Approximately, 63% of the specimens are georeferenced. The identification of the specimens in the collection has been carried out by the plant biology department at the University of Malaga and plus 40% of the specimens has been reviewed by experts. The MGC-Cormof dataset has been revised by DarwinTest v3.2 tool (Ortega-Maqueda and Pando 2008) before being published in GBIF. The data included in this database are important for conservation works, taxonomy, flora, cartography, phenology, palynology, among others.

El Herbario de la Universidad de Málaga (Herbario MGC) está constituido por cuatro colecciones biológicas. La colección de plantas vasculares (MGC Cormof) es la colección principal del herbario. La base de datos MGC-Cormof tiene como objetivo la digitalización y publicación de los datos asociados con los más de 76.000 ejemplares depositados en la colección, de los cuales el 97,2% de las muestras se encuentran identificadas a nivel de especie. Desde 2011, los Servicios Centrales de Investigación (SCAI) de la Universidad de Málaga son responsables de mantener el herbario y sus respectivas bases de datos. Esta colección está en continuo crecimiento, con una incorporación anual de unos 1.500 ejemplares. Casi el 96% de la colección está digitalizada, a través del programa Herbar v3.7.1 (F. Pando et al. 1996–2011) por lo que más de 73.000 especímenes son accesibles a través de la red de GBIF (http://data.gbif.org/datasets/resource/8105/). Actualmente, la colección MGC-Cormof está constituida por 247 familias y 8.110 taxones, distribuidos en angiospermas (93,97%), helechos y plantas afines (4,89%) y gimnospermas (1,14%). Las familias y géneros mejor representados en la colección son Compositae, Leguminosae, Gramineae, Labiatae, Caryophyllaceae, Teucrium, Silene, Asplenium, Linaria y Quercus. La mayoría de los especímenes provienen de la región del Mediterráneo Occidental, fundamentalmente del sur de España (Andalucía: 82% de las muestras) y del norte de Marruecos (2,17%). Aproximadamente, el 63% de las muestras se encuentran georreferenciadas. La identificación de los ejemplares de la colección ha sido realizada por personal del departamento de biología vegetal de la Universidad de Málaga y además un 40% de los ejemplares ha sido revisado por especialistas. La base de datos MGC-Cormof ha sido revisada mediante la herramienta DarwinTest v3.2 (Ortega-Maqueda and Pando 2008) antes de ser publicada en GBIF. Los datos incluidos en esta base de datos son importantes para trabajos de conservación, taxonomía, flora, cartografía, fenología, palinología, entre otros.

## General description

The MGC-Cormof dataset belongs to the University of Malaga MGC Herbarium, and has been the responsibility of the Central Research Service (SCAI) of the same university since 2011. In addition to that of MGC-Cormof, the MGC Herbarium contains three other datasets, which are not the subject of this paper: MGC-Algae (5.400 sheets), MGC-Briof (1.850 sheets) and MGC-Lichen (350 sheets). The MGC-Cormof collection has nearly 76.000 sheets, of which 97.2% are identified at species level. Most of the plant specimens are collected from Andalusia (Southern Spain) with 60.456 sheets, which Malaga province is the most important area represented (39.902 sheets). In addition, the herbariun contains plant specimens from Northern Morocco, several other places in Spain, and countries from Europe, Africa and South America

The herbarium collections are the result of several research projects that have been carried out over the last 40 years. This collections are very active and in continual growth, with an annual intake of about 1.500 specimens. The main data contributors are researchers from the Botany Area of the Plant Biology Department of the University of Malaga, which was responsible for administering the herbarium until 2011. Ninety-six percent of the collection is digitalized, by Herbar v3.7.1 software (F. Pando et al. 1996–2011),and so the dataset has 73.156 specimens, which are available on the GBIF data portal (http://data.gbif.org/datasets/resource/8105/).

MGC herbarium is one of the reference herbaria for Flora Iberica (Castroviejo 1986–2012) and Vascular Flora in Eastern Andalusia (Flora Vascular de Andalucía Oriental) (Blanca et al. 2009). The journal Acta Botanica Malacitana (B. Cabezudo 1975–2013) (http://www.biolveg.uma.es/abm/abm.html) is closely associated with the MGC Herbarium and periodically publishes papers that are based on data included in its dataset.

## Project details

Specific projects for computerizing the herbarium specimens, a task that began in 2006, are mentioned below:

**Project title:** “Informatización del Herbario de la Universidad de Málaga (MGC)”.

**Project personnel:** Dr. Baltasar Cabezudo Artero

**Funding:** Digitalisation supported by the Spanish Ministry of Education and Science (ref. CLEG2004-21156-E).

**Length:** January 2006–September 2009.

**Project title:** “Realización de una prestación de servicios para el mantenimiento e inclusión de pliegos del proyecto Flora de Andalucía Oriental en el Herbario público de la Universidad de Málaga”.

**Project personnel:** Dr. Baltasar Cabezudo Artero

**Funding:** Digitalisation supported by the Regional Government of Andalusia (Junta de Andalucía) (ref. 8.06/03.3353).

**Length:** February 2010–February 2011.

**Project title:** “Digitalización y Gestión de las Bases de Datos del Herbario MGC de la Universidad de Málaga”.

**Project personnel:** Dr. José García Sánchez

**Funding:** Digitalisation supported by Ministry for Science and Innovation (ref. PTA2011-6046-I).

**Length:** January 2012–December 2014.

Temporary staff for assisting in the in fieldwork were contracted through the Regional Government of Andalusia, Junta de Andalucía (Spain) research group: Biodiversidad, Conservación y Recursos Vegetales; Group code: RNM-115.

## Data published through GBIF:

Data from the MGC-Cormof dataset can be consulted in the Spanish GBIF Node IPT and the data resource of the University of Malaga.

http://www.gbif.es:8080/ipt/resource.do?r=mgc_cormof

http://data.gbif.org/datasets/resource/8105/

## Taxonomic coverage

### General taxonomic coverage:

**Figure 1.**
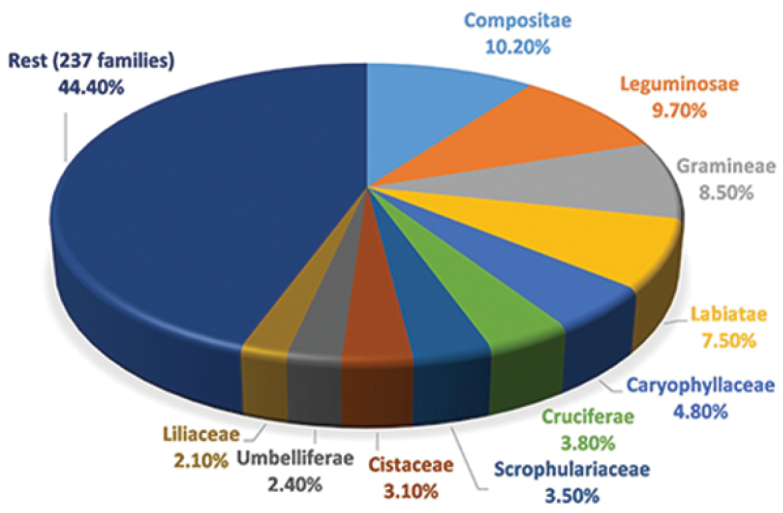
Main families in MGC-Cormof collection

**Figure 2.**
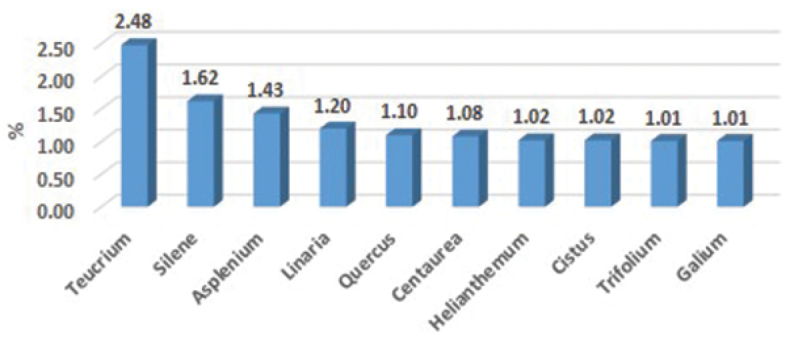
Main genera in MGC-Cormof collection.

The 76.000 sheets of the MGC-Cormof collection are grouped into 241 families (Figure 1), 1.551 genera (Figure 2), 6.158 species and 8.110 taxa (including infraspecific categories). Most of the specimens are angiosperms (93.97%), followed by ferns and fern allies (4.89%) and gymnosperms (1.14%). The main families in order of abundance are Compositae (10.2%), Leguminosae (9.7%), Gramineae (8.5%), Labiatae (7.5%), Caryophyllaceae (4.8%), Cruciferae (3.8%), Scrophulariaceae (3.5%), Cistaceae (3.1%), Umbelliferae (2.4%) and Liliaceae (2.1%). Main genera in order of abundance are Teucrium (2.48%), Silene (1.62%), Asplenium (1.43%), Linaria (1.20%), Quercus (1.10%), Centaurea (1.08%), Helianthemum (1.02%), Cistus (1.02%), Trifolium (1.01%) and Galium (1.01%).

It is important to mention that this collection contains a wide representation of plants from several Protected Areas of southern Spain (Cabezudo et al. 2005; Pérez Latorre et al. 1998, 1999, 2002), endemic species (Blanca et al. 2009; Salvo et al. 1983), threatened species (Blanca et al. 1999, 2000, 2009; Cabezudo et al. 2005; Salvo et al. 1983), invasive species (Dana et al. 2005), ornamental species and many weeds.

### Taxonomic ranks

**Kingdom:** Plantae

**Phylum:** Pteridophyta, Spermatophyta

**Class:** Magnoliopsida, Liliopsida, Filicopsida, Lycopsida, Coniferopsida, Equisetopsida, Ophioglossopsida, Gnetopsida, Taxopsida, Cycadopsida, Gingkgopsida, Psilotopsida.

**Family:** Acanthaceae, Aceraceae, Adiantaceae, Agavaceae, Aizoaceae, Alismataceae, Alliaceae, Amaranthaceae, Amaryllidaceae, Anacardiaceae, Anemiaceae, Annonaceae, Apocynaceae, Aquifoliaceae, Araceae, Araliaceae, Araucariaceae, Aristolochiaceae, Asclepiadaceae, Aspidiaceae, Aspleniaceae, Athyriaceae, Azollaceae, Balsaminaceae, Bartramiaceae, Basellaceae, Begoniaceae, Berberidaceae, Betulaceae, Bignoniaceae, Blechnaceae, Bombacaceae, Boraginaceae, Botrychiaceae, Bromeliaceae, Buddlejaceae, Butomaceae, Buxaceae, Cactaceae, Callitrichaceae, Calyceraceae, Campanulaceae, Cannaceae, Capparaceae, Caprifoliaceae, Caricaceae, Caryophyllaceae, Casuarinaceae, Cecropiaceae, Celastraceae, Cephalotaxaceae, Ceratophyllaceae, Chenopodiaceae, Cistaceae, Clethraceae, Clusiaceae, Cneoraceae, Combretaceae, Commelinaceae, Compositae, Convallariaceae, Convolvulaceae, Coriariaceae, Cornaceae, Corylaceae, Crassulaceae, Cruciferae, Cryptogrammaceae, Cucurbitaceae, Culcitaceae, Cunoniaceae, Cupressaceae, Cyatheaceae, Cycadaceae, Cynomoriaceae, Cyperaceae, Davalliaceae, Dennstaedtiaceae, Dicksoniaceae, Dioscoreaceae, Dipsacaceae, Dracaenaceae, Droseraceae, Dryopteridaceae, Ebenaceae, Elaeagnaceae, Elatinaceae, Empetraceae, Ephedraceae, Equisetaceae, Ericaceae, Eriocaulaceae, Erythroxylaceae, Escalloniaceae, Euphorbiaceae, Fagaceae, Flacourtiaceae, Frankeniaceae, Gentianaceae, Geraniaceae, Gesneriaceae, Ginkgoaceae, Gleicheniaceae, Globulariaceae, Gramineae, Grammitidaceae, Grossulariaceae, Guttiferae, Haloragaceae, Hamamelidaceae, Hemionitidaceae, Hippochaetaceae, Hippuridaceae, Hydrangeaceae, Hydrocharitaceae, Hydrocotylaceae, Hydrophyllaceae, Hymenophyllaceae, Hypolepidaceae, Iridaceae, Isoetaceae, Juglandaceae, Juncaceae, Juncaginaceae, Labiatae, Lauraceae, Leguminosae, Lemnaceae, Lentibulariaceae, Liliaceae, Linaceae, Lycopodiaceae, Lythraceae, Magnoliaceae, Malpighiaceae, Malvaceae, Marsileaceae, Melastomataceae, Meliaceae, Melianthaceae, Menispermaceae, Molluginaceae, Monimiaceae, Monotropaceae, Moraceae, Musaceae, Myoporaceae, Myricaceae, Myrsinaceae, Myrtaceae, Najadaceae, Nephrolepidaceae, Neuradaceae, Nyctaginaceae, Nymphaeaceae, Oleaceae, Oleandraceae, Onagraceae, Ophioglossaceae, Orchidaceae, Orobanchaceae, Osmundaceae, Oxalidaceae, Paeoniaceae, Pandanaceae, Papaveraceae, Parnassiaceae, Passifloraceae, Phormiaceae, Phyladelphyae, Phytolaccaceae, Pinaceae, Piperaceae, Pittosporaceae, Plantaginaceae, Platanaceae, Plumbaginaceae, Podocarpaceae, Polemoniaceae, Polygalaceae, Polygonaceae, Polypodiaceae, Portulacaceae, Posidoniaceae, Potamogetonaceae, Primulaceae, Proteaceae, Psilotaceae, Pteridaceae, Punicaceae, Pyrolaceae, Rafflesiaceae, Ranunculaceae, Resedaceae, Rhamnaceae, Rosaceae, Rubiaceae, Ruppiaceae, Rutaceae, Salicaceae, Salviniaceae, Santalaceae, Sapindaceae, Sapotaceae, Saxifragaceae, Scrophulariaceae, Selaginellaceae, Simaroubaceae, Sinopteridaceae, Smilacaceae, Solanaceae, Sparganiaceae, Sterculiaceae, Strelitziaceae, Styracaceae, Symplocaceae, Tamaricaceae, Taxaceae, Taxodiaceae, Theligonaceae, Thymelaeaceae, Thymeleaceae, Tiliaceae, Tropaeolaceae, Typhaceae, Ulmaceae, Umbelliferae, Urticaceae, Valerianaceae, Verbenaceae, Violaceae, Viscaceae, Vitaceae, Vivianiaceae, Winteraceae, Woodsiaceae, Zamiaceae, Zannichelliaceae, Zingiberaceae, Zosteraceae, Zygophyllaceae

### Common names

Tracheophytes, Cormophytes, Vascular Plants, Sunflowers, Legumes, True Grasses, Mints, Pinks, Crucifers or Mustard, Figworts, Rock Roses, Parsley or Carrot family, Lyli family, Germander, Campion, Petako Rauriki, Spurred Snapdragon, Oak, Knapweed, Rock Rose, Rock Rose, Clover, Bedstraw

## Spatial coverage

### General spatial coverage

**Figure 3.**
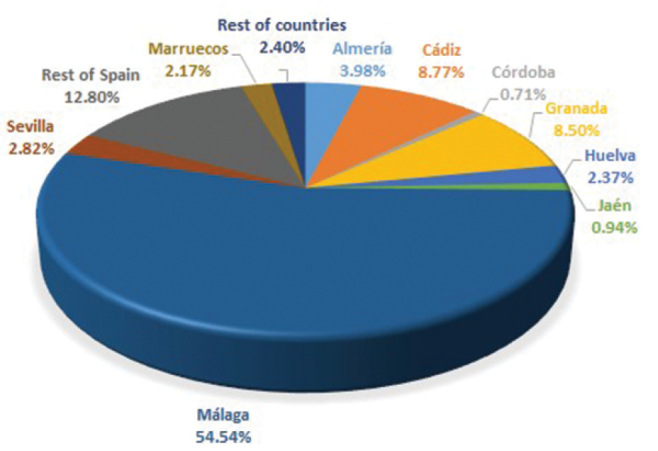
Spatial coverage of MGC-Cormof collection.

**Figure 4.**
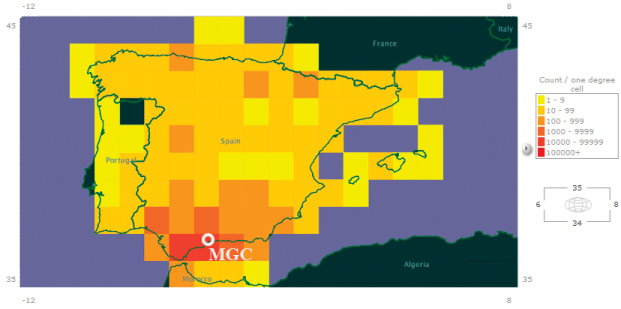
Distribution map of the records from the MGC-Cormof dataset. This map shows most of the records in the dataset (Iberian Peninsula and North of Morocco). Source: GBIF Data Portal (http://data.gbif.org/datasets/resource/8105/).

Most of the data refer to the Western Mediterranean Region, mainly Southern Spain (Andalusia 82% of sheets) and Northern Morocco (2.17%). Andalusia is composed of 8 provinces, Malaga province being the most important with 53% of sheets, followed by Cádiz (8.77%) and Granada (8.50%). Moreover, 11% of the data refer to the rest of Spain and 7% from 50 countries of Europe, Africa and South America mainly (Figures 3 and 4).

The MGC-Cormof collection has a large number of plants from all the main Protected Areas of Malaga province, including Natural Parks (Sierra de las Nieves, Sierras de Tejeda, Almijara y Alhama, and Montes de Málaga) and Natural Areas (Los Reales de Sierra Bermeja, Torcal de Antequera, and Desfiladero de los Gaitanes), as well as other Protected Areas of southern Spain, some of which are Natural Parks shared with Malaga (e.g. Los Alcornocales, Sierra de Grazalema) and the Protected Landscape Corredor Verde (Green Corridor) del Guadiamar. Table 1 shows the approximate number of sheets from these protected areas. Moreover, many plants considered as agricultural weeds and others taken from roads and cities as well as ornamental plants (910 sheets) from public parks and gardens of the city of Malaga, are included. Sixty-three percent of the specimens are georeferenced. All of them have been referenced by MGRS coordinate system, which have been transformed into geographical coordinate before uploading to the GBIF Portal by Herbar 3.7.1 software (Pando et al. 1996–2011). The accuracy of the coordinate grids in MGRS system varies from 1 m^2^ to 10 km^2^ and the accuracy in geographical coordinate varies from 1 to 7071 m^2^.

**Table 1.** Number of sheets from main protected areas represented in the herbarium.<br/>

**Protected area**	**Nº sheets**
Natural Park Sierras de Tejeda, Almijara y Alhama	8890
Natural Park Sierra de las Nieves	2545
Natural Park Sierra de Grazalema	1185
Natural Park Los Alcornocales	940
Natural Park Montes de Málaga	680
Natural Area Los Reales de Sierra Bermeja	1700
Natural Area Torcal de Antequera	1180
Natural Area Desfiladero de los Gaitanes	425
Protected Landscape Corredor Verde del Guadiamar	910

### Coordinates

34°0'0"S and 51°42'0"N; 116°2'60"W and 83°25'48"E

## Temporal coverage

1837–2012.

**Figure 5.**
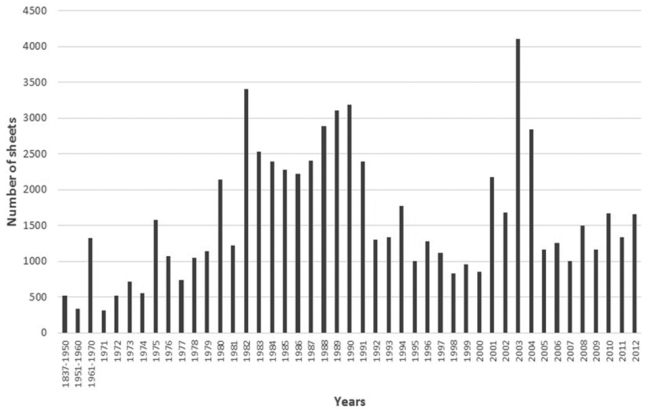
Number of sheets gathered between 1837 and 2012 in the MGC-Cormof collection.

Figure 5 represents the year of gather of the sheets incorporated in the MGC-Cormof collection. The sheets prior to 1972 (date of the creation of the MGC Herbarium) and also some of them, along the life of the herbarium, are the result of donations and exchanges with several herbaria. The best represented are The Real Jardín Botánico de Madrid Herbarium (MA Herbarium), Barcelona Botanical Institute Herbarium (BC Herbarium), University of Seville Herbarium (SEV Herbarium), University of Granada Herbarium (GDA Herbarium) and University of Extremadura Herbarium (UNEX Herbarium). The differences observed in the number of sheets along the time are mainly due to develop of research works and post grade studies carried out in the Botany Area of the Department of Plant Biology at University of Malaga.

## Natural collections description

*Collection name*: MGC-Cormof

*Collection identifier*: 962cceea-f762-11e1-a439-00145eb45e9a

*Formation period*: 1972-2013

*Specimen preservation method*: Dried and pressed

*Curatorial unit 1*: 73.156 with an uncertainty of 0 (Sheets)

*Curatorial unit 2*: 6.158 with an uncertainty of 0 (Species)

*Curatorial unit 3*: 1.551 with an uncertainty of 0 (Genera)

*Curatorial unit 4*: 247 with an uncertainty of 0 (Families)

## Methods

### Study extent

Most plants are from Southern Spain (Andalusia), Malaga province being the most widely represented area, the aim being to cover the widest degree of plant biodiversity for this territory. In addition, the collection contains plants from Northern Morocco and several places from the rest of Spain and countries from Europe, Africa and South America.

### Samplingdescription

The plants of this collection were mainly gathered by researchers of the Botany Area of the Department of Plant Biology at University of Malaga, as well as by members of the herbarium. A small component of the collection comes from exchanges or donations from other research centres or researchers.

### Method step description

Before incorporating new plants in the herbarium, the steps described below are followed.

First, the material is pressed and dried, mounted on double A2 standard size (42 × 59.4 cm) sheets which perfectly cover and protect the specimen. Inside each sheet, an identification label provides the following information: taxonomy, country, province, county, locality, georeference, date, ecology, collectors and determinations. To kill any insects contained in the sheets, they are frozen at -20 °C for 72 hours. Periodically, the herbarium room is fumigated. The specimens are kept in compact shelving cabinets and arranged taking into account three main taxonomic groups: pteridophytes, gymnosperms and angiosperms. Within each group, the specimens are alphabetically arranged by families, genera and species.

### Quality control

Every specimen of the MGC-Cormof collection has been identified by researchers of the Botany Area of the Department of Plant Biology at University of Malaga. Moreover, 40% of the specimens of this collection have subsequently been taxonomically revised for regional or national studies of flora or taxonomical revisions. Each taxonomic modification is incorporated into the database.

The dataset is analysed in search of digitalisation errors before uploading to the GBIF Portal. This check is carried out by the DarwinTest v3.2 tool (Ortega-Maqueda and Pando 2008), provided by the Spanish GBIF Node. This tool looks for mistakes in taxonomy, dates, geospatial information, collectors, identifiers, etc.

## Datasets

### Dataset description

**Object name:** Darwin Core Archive MGC Herbarium of the University of Malaga (Spain): MGC-Cormof dataset

**Character encoding:** UTF-8

**Format name:** Darwin Core Archive format

**Format version:** 1.0

**Distribution:** http://www.gbif.es:8080/ipt/resource.do?r=mgc_cormof

**Date of metadata creation:** 2013-04-04

**Metadata language:** English

**Hierarchy level:** Dataset

**User license:** The MGC-Cormof dataset is made available under the Open Data Commons Attribution License: http://www.opendatacommons.org/licenses/by/1.0/

**DarwinCore elements:** Thirty-two (32) DarwinCore elements (http://purl.org/dc/terms/) included in the dataset published through the GBIF network. These are: id, preparations, typeStatus, eventDate, family, specificEpithet, minimumElevationInMeters, decimalLongitude, identifiedBy, occurrenceRemarks, dateIdentified, individualCount, collectionCode, minimumDepthInMeters, kingdom, coordinateUncertaintyInMeters, infraspecificEpithet, institutionCode, country, stateProvince, modified, recordedBy, genus, cientificNameAuthorship, maximumDepthInMeters, locality, recordNumber, catalogNumber, scientificName, maximumElevationInMeters, decimalLatitude, basisOfRecord

## External datasets

### Dataset description

**Object name:** Universidad de Málaga: MGC-Cormof

**DiGIR information:** http://gbif.cie.uma.es/digir/DiGIR.php

**Character encoding:** iso-8859-1

**Format name:** Darwin Core

**Format version:** 1.2

**Distribution:** http://data.gbif.org/datasets/resource/8105/

**Date of metadata creation:** 2009-02-19

**Metadata language:** English

**Hierarchy level:** Dataset
